# Functional inactivation of *OsGCNT* induces enhanced disease resistance to *Xanthomonas oryzae* pv. *oryzae* in rice

**DOI:** 10.1186/s12870-018-1489-9

**Published:** 2018-11-01

**Authors:** Xia Xu, Zheng Chen, Yong-feng Shi, Hui-mei Wang, Yan He, Lei Shi, Ting Chen, Jian-li Wu, Xiao-bo Zhang

**Affiliations:** State Key Laboratory of Rice Biology, Chinese National Center for Rice Improvement, China National Rice Research Institute, Hangzhou, 310006 China

**Keywords:** *Oryza sativa* L., Spotted-leaf, *OsGCNT*, Premature leaf senescence, Defense response

## Abstract

**Background:**

Spotted-leaf mutants are important to reveal programmed cell death and defense-related pathways in rice. We previously characterized the phenotype performance of a rice spotted-leaf mutant *spl21* and narrowed down the causal gene locus *spl21*(*t*) to an 87-kb region in chromosome 12 by map-based cloning.

**Result:**

We showed that a single base substitution from A to G at position 836 in the coding sequence of *Oryza sativa beta-1,6-N-acetylglucosaminyl transferase* (*OsGCNT*), effectively mutating Tyr to Cys at position 279 in the translated protein sequence, was responsible for the spotted-leaf phenotype as it could be rescued by functional complementation. Compared to the wild type IR64, the spotted-leaf mutant *spl21* exhibited loss of chlorophyll, breakdown of chloroplasts, down-regulation of photosynthesis-related genes, and up-regulation of senescence associated genes, which indicated that *OsGCNT* regulates premature leaf senescence. Moreover, the enhanced resistance to the bacterial leaf blight pathogen *Xanthomonas oryzae* pv. *oryzae*, up-regulation of pathogenesis-related genes and increased level of jasmonate which suggested that OsGCNT is a negative regulator of defense response in rice. *OsGCNT* was expressed constitutively in the leaves, sheaths, stems, roots, and panicles, and OsGCNT-GFP was localized to the Golgi apparatus. High throughput RNA sequencing analysis provided further evidence for the biological effects of loss of *OsGCNT* function on cell death, premature leaf senescence and enhanced disease resistance in rice. Thus, we demonstrated that the novel OsGCNT regulated rice innate immunity and immunity-associated leaf senescence probably by changing the jasmonate metabolic pathway.

**Conclusions:**

These results reveal that a novel gene *Oryza sativa beta-1,6-N-acetylglucosaminyl transferase* (*OsGCNT*) is responsible for the spotted-leaf mutant *spl21,* and OsGCNT acts as a negative-regulator mediating defense response and immunity-associated premature leaf senescence probably by activating jasmonate signaling pathway.

**Electronic supplementary material:**

The online version of this article (10.1186/s12870-018-1489-9) contains supplementary material, which is available to authorized users.

## Background

Programmed cell death (PCD) refers to the genetically controlled death of a cell [1]. It is well known that PCD plays a fundamental role in varieties of biological functions including innate immunity in plants. Plants have evolved complicated mechanisms to defend themselves from pathogen infections [2]. The hypersensitive response (HR), a type of PCD, is the most common characteristic of plant disease resistance, which triggers rapid cell death to inhibit further invasion of pathogens in host plant tissues [3]. Lesion mimic mutants (LMMs) or the specifically termed spotted-leaf (spl) mutants in rice could produce necrotic lesions similar to that caused by HR without pathogen infection, abiotic stress or mechanical damage [4]. In fact, it has been reported that numerous rice spl mutants display significantly enhanced disease resistance to multiple pathogens [5, 6]. The identification and characterization of novel spl mutants would facilitate the elucidation of mechanisms involved in plant innate immunity.

Leaf senescence, as the last stage of leaf development, is an important biological process [7]. However, premature leaf senescence affects crop yield and biomass production [8]. It has been observed that many spl mutants exhibit spontaneous cell death in pre-senescent green leaves under normal growth conditions [7]. In recent years, many spl mutants have been identified and the causal genes have been cloned. In rice, these genes encode various types of proteins such as zinc finger protein [9], extracellular leucine-rich repeat (eLRR) domain protein [10], hypersensitive induced reaction protein [11], clathrin-associated adaptor protein [12], coproporphyrinogen III oxidase [13], nucleotide-binding site containing protein leucine-rich repeat (NBS-LRR) protein [14, 15], splicing factors [16, 17], mitogen-activated protein kinase kinase kinase (MAPKKK) [18, 19], E3 ubiquitin ligases [20–22], UDP-N-acetylglucosamine pyrophosphorylase [23], AAA-type ATPase [2, 24], eukaryotic translation elongation factor [3, 25], subunit of the light-harvesting complex I [26], and proteins involved in biochemical pathways of eukaryotic release factor 1 responsible for salicylic acid (SA)-dependent defense response [27]. The wide range of protein categories indicates leaf senescence is not only important but also a complicated biological process. Nevertheless, the identification and characterization of more novel spl mutants may be helpful to elucidate the mechanism of immunity-associated leaf senescence in plants.

Glycosyltransferases (GTs; EC 2.4.x.y) belong to a superfamily, and their biochemical reactions are associated with the formation of glycosidic bonds through the transfer of sugars from activated donor molecules to a wide range of lipophilic small molecule acceptors including lipids, proteins, hormones, secondary metabolites, and oligosaccharides [28]. It has been reported that GTs are involved in many biological processes including metabolic regulation, synthesis of diverse secondary metabolites, modification of hormones and cell wall synthesis [29]. In CAZy (Carbohydrate Active Enzyme) database, rice genome contains 609 potential GT members which have been grouped into 40 gene families. The *beta*-galactoside *beta*-1-6- and *beta*-1-3-N-acetylglucosaminyltransferases (*beta*-1-6GnT and *beta*-1-3GnT) that synthesize blood group I and i antigens were first identified in rat tissues [30]. In human, *beta*-1,6-N-acetylglucosaminyltransferase is classified into two types, the core 2-branching enzyme and I-branching enzyme. It has been found that *Core 2 beta-1,6-N-acetylglucosaminyltransferase1* (*GCNT1*) expression in prostate biopsy specimen is an indicator of prostate cancer aggressiveness [31], and *I-branching glucosaminyl (N-acetyl) transferase 2* (*GCNT2*) expression is also significantly correlated to the metastatic phenotype in breast tumor samples [32]. However, the function of GCNT in plants is still largely unknown.

In our previous study, we isolated the rice spotted-leaf mutant *spl21* and mapped the causal gene locus *spl21*(*t*) to an 87-kb region in chromosome 12 [33]. Here, we show that *spl21*(*t*) (*LOC_Os12g42420*) encodes a novel *Oryza sativa* beta-1,6-N-acetylglucosaminyl transferase (OsGCNT). Functional complementation, high-throughput mRNA sequencing (RNA-seq), plant hormone level determination, and gene functional analysis suggest that a single base substitution in *OsGCNT* is responsible for the spotted-leaf phenotype including defense response and premature leaf senescence in rice. These results indicate that loss of *OsGCNT* function is associated with programmed cell death and enhances disease response probably by activating the jasmonate signaling pathway.

## Results

### Map-based cloning of *OsGCNT*

**Fig. 1:**
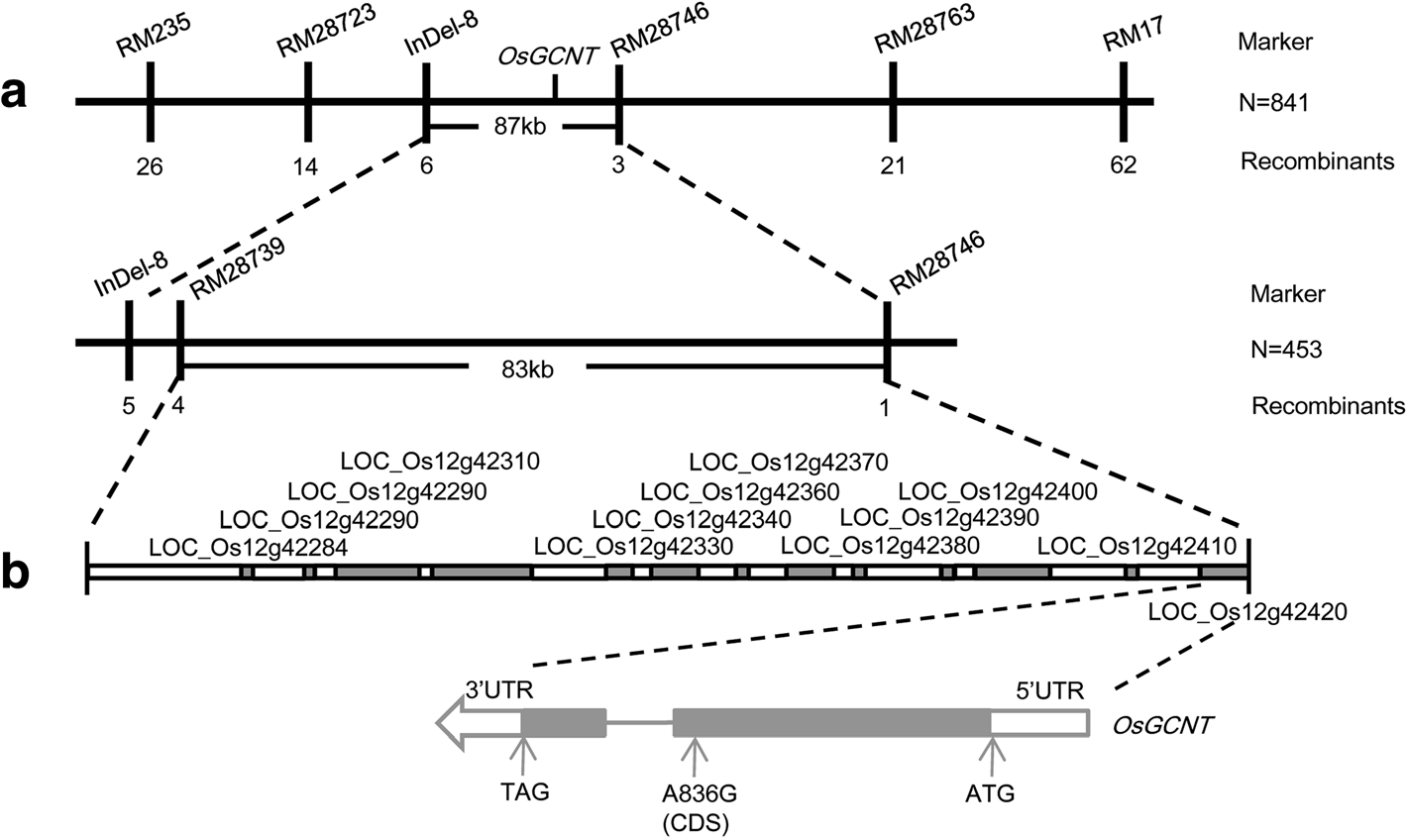
Map-based cloning of *OsGCNT*. **a** *OsGCNT* locus is further refined to a 83-kb region between marker RM28746 and RM28739. **b** Single base substitution at position 836 (A836G, Y279C)

The *spl21*(*t*) locus was previously delimited to an 87 kb region in rice chromosome 12 [33]. To fine-map the locus, an additional 453 F_2_ individuals with the spotted-leaf phenotype were genotyped and the *spl21*(*t*) locus was further refined to an 83 kb region spanning 13 ORFs (Fig. 1a). Sequence comparison of those ORFs cloned from both the mutant *spl21* and WT, we detected only one nonsynonymous mutation (A836G, Y279C) in the ORF *LOC_Os12g42420*. The other 12 ORFs did not have any mutation compared to their respective wild-type loci (Fig. 1b). The 2465 bp *LOC_Os12g42420* contains 2 exons, 1 intron, and thus was considered the most likely candidate gene responsible for the spotted-leaf phenotype.

**Fig. 2:**
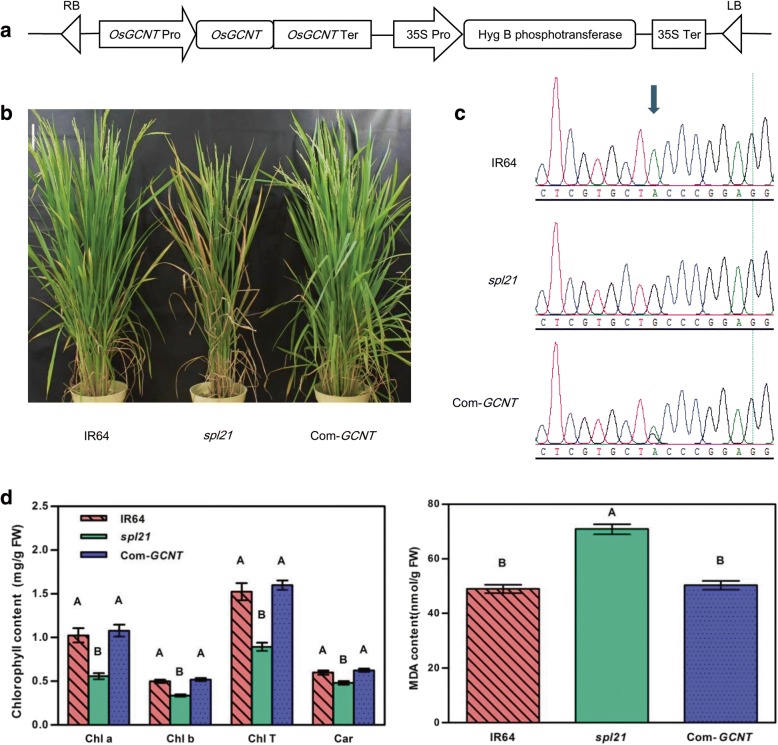
*OsGCNT* complements the *spl21* spotted-leaf phenotype. **a** Complementation constructs pC1300-C. **b** Phenotype of IR64, *spl21*, and complementation transgenic line (Com-*GCNT*). **c** Sequencing analysis of complementation transgenic lines. Arrowhead indicates the mutation site. **d** The contents of Chlorophyll and MDA in IR64, *spl21*, and complementation lines. Data are means ± SD of three biological replicates. Means with different letters are significantly different at *P* ≤ 0.01 using Duncan’s test

To verify whether the single base substitution in *LOC_Os12g42420* was responsible for the *spl21* phenotype, the complementary vector pC1300-C harboring the wild type allele was introduced into the mutant embryogenic calli by *Agrobacterium tumefaciens-*mediated transformation. A total of 28 positive independent T_0_ transformants were obtained and all exhibited the normal green leaf color without lesions throughout their entire growth period. The results demonstrated that the WT allele could rescue the spotted-leaf phenotype and was indeed the target gene (Fig. 2a-c). Furthermore, *spl21* shows the low level of chlorophyll contents at different leaf developmental stages and a high level of H_2_O_2_ accumulation at/around leaf lesions with a high malonaldehyde (MDA) content compared to WT [33]. We measured the contents of chlorophyll a (Chl a), chlorophyll b (Chl b), total of chlorophyll (Chl T), carotenoids (Car) and MDA in the complemented plants to determine whether they were restored to the wild-type levels. The MDA contents in both the complementation lines and WT plants were similar and significantly lower than that of *spl21*. While the contents of Chl a, Chl b and Car in complementation lines and WT were similar but significantly higher than that of *spl21*, indicating that they recovered to the level of WT (Fig. 2d). Taken together, our results demonstrated that *LOC_Os12g42420* was the target gene responsible for the spotted-leaf phenotype of *spl21*.

According to the sequence comparison between genomic DNA and cDNA, *LOC_Os12g42420* is composed of 2 exons separated by 1 intron (Fig. 1b). According to the rice genome annotation database (http://rice.plantbiology.msu.edu), the coding sequence (CDS) of *LOC_Os12g42420* consists of 1098 nucleotides, and putatively encodes a protein of 365 amino acid residues with a molecular mass of approximately 41 kD. *LOC_Os12g42420* is predicted to encode a putative DNA binding protein which contains a Branch domain spanning the amino acid residues from 82 to 332. The Branch domain-containing proteins consist of two types of *beta*-1,6-N-acetylglucosaminyl transferase enzymes (I-branching enzyme and core-2 branching enzyme), thus *LOC_Os12g42420* is supposed to encode a novel *beta*-1,6-N-acetylglucosaminyl transferase and we designate the locus as *Oryza sativa beta-1,6-N-acetylglucosaminyl transferase* (*OsGCNT*).

**Fig. 3:**
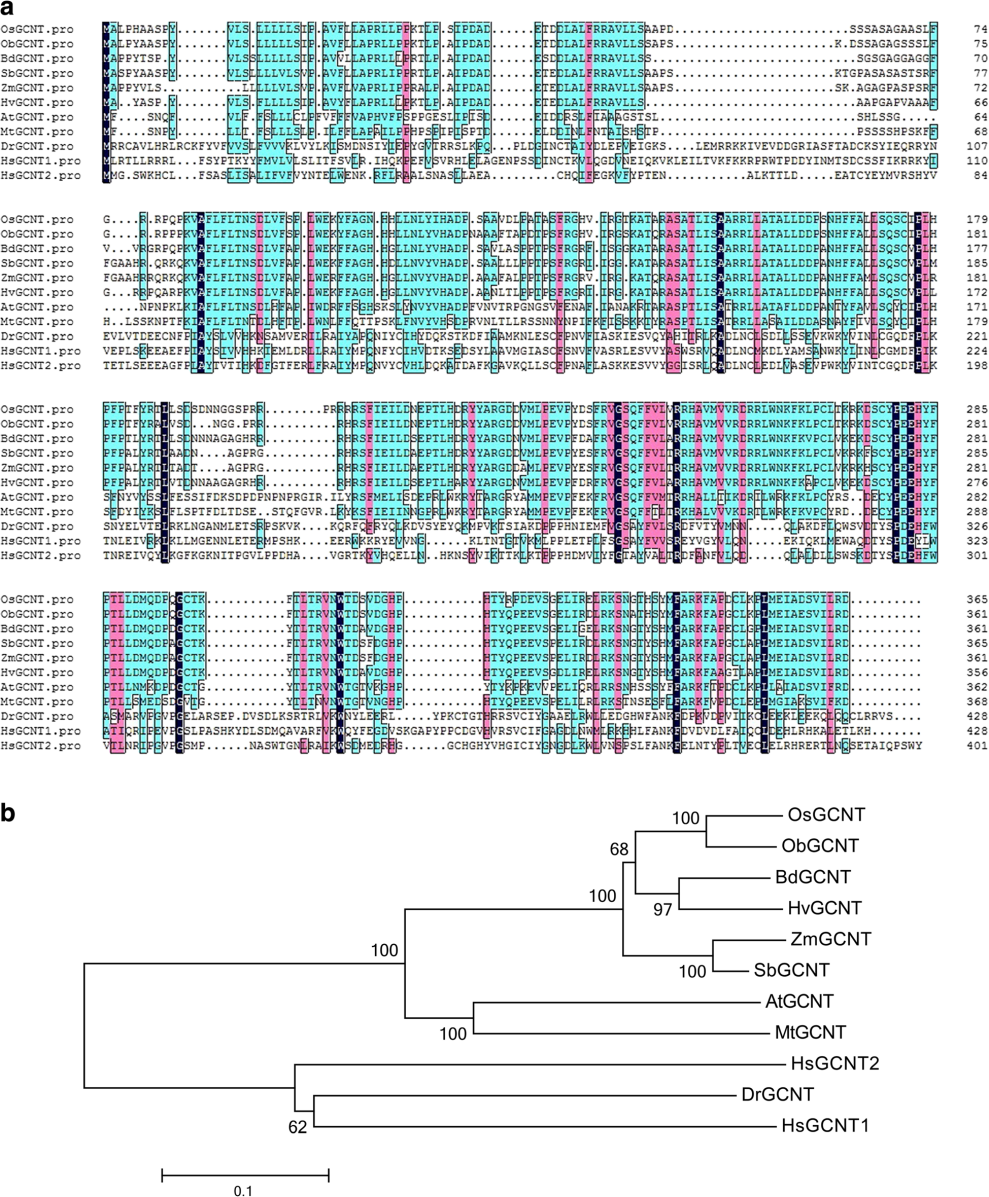
Sequence alignment and phylogenetic analysis of OsGCNT with its homologues. **a** OsGCNT shares high identity with its homologues, shown by the amino acids highlighted in black. **b** Phylogenetic analysis of proteins from different organisms. Phylogenetic tree was constructed by Neighbor-joining method using the MEGA program. Bootstrap values from 100 replicates are indicated at each node. Bar represents the number of amino acid differences per site. All GCNT proteins used and their GenBank accessions are as follows: *Oryza sativa* OsGCNT (MH181877), *Oryza brachyantha* ObGCNT (XP_006664213.1), *Arabidopsis thaliana* AtGCNT (NP_680193.2), *Brachypodium distachyon* BdGCNT (XP_003577263.1), *Zea mays* ZmGCNT (XP_008674099.1), *Sorghum bicolor* SbGCNT (XP_002443582.1), *Medicago truncatula* MtGCNT (XP_003596911.1), *Hordeum vulgare* HvGCNT (BAJ85768.1), *Danio rerio* DrGCNT (NP_963877.1), *Homo sapiens* HsGCNT1 (NP_001481.2) and *Homo sapiens* HsGCNT2 (NP_663624.1). Distance scale =0.1

There is only one copy of *OsGCNT* in the rice genome. The *OsGCNT* homologous genes were identified in 9 species by a BLAST search (Fig. 3a). We then assessed the evolutionary relationships between OsGCNT and its homologues in different species by constructing a bootstrap consensus phylogenetic tree using the neighbour-joining method (Fig. 3b). Sequence comparison indicates that OsGCNT is most comparable to gramineae homologs such as ObGCNT (89%), BdGCNT (80%), SbGCNT (80%), HvGCNT (80%) and ZmGCNT (79%) while OsGCNT has a high level of similarity to the dicot AtGCNT (69%) and a lower similarity to the human HsGCNT1 (20.2%) (Fig. 3). Notably, the amino acid mutation (Y279C) in rice is located in the Branch domain, and the corresponding site is conserved among different species. The results indicate that *OsGCNT* is structurally conserved and that the Y279C mutation is likely critical to its biological function.

### *OsGCNT* is expressed constitutively and OsGCNT-GFP probably localizes to the Golgi apparatus

**Fig. 4:**
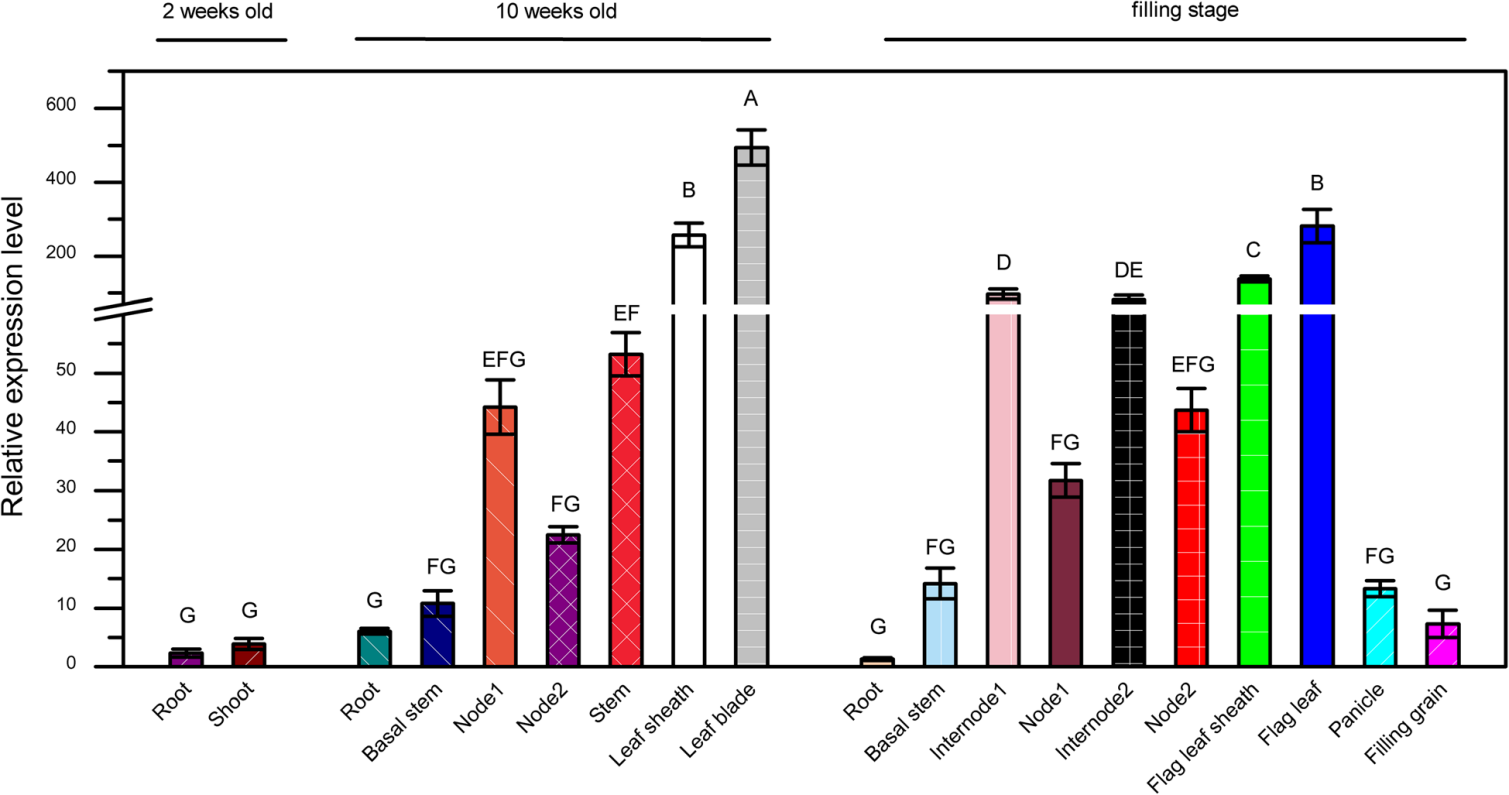
Quantitative PCR analysis of wild-type *OsGCNT* mRNA expression. Relative expression levels of *OsGCNT* in various rice tissues at different growth stages. Data are means ± SD of three biological replicates. Means with different letters are significantly different at *P* ≤ 0.01 using Duncan’s test

To determine the expression pattern of *OsGCNT*, we performed qRT-PCR analysis on the total RNA of roots, stems, nodes, internodes, leaves, leaf sheaths, panicles, and filling grains from WT. *OsGCNT* was expressed in all the organs examined. The highest expression was detected in leaves and leaf sheaths at 10 weeks old stage, with relatively weaker expression in roots and stems. Similar tendency of expression levels were observed at the grain filling stage (Fig. 4). These results indicated that *OsGCNT* was constitutively expressed at all developmental stages of rice.

**Fig. 5:**
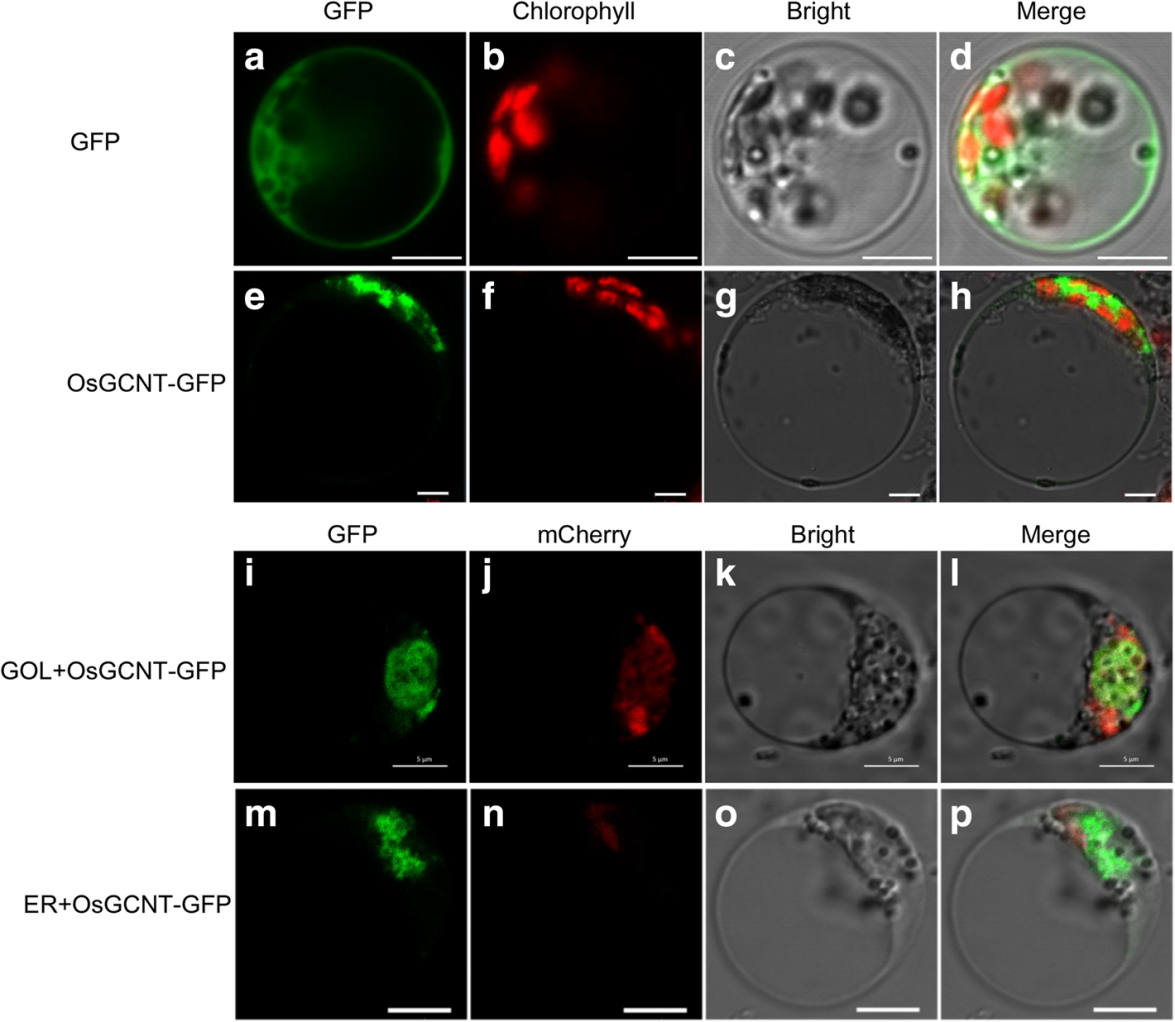
Subcellular location of OsGCNT-GFP in rice protoplasts. **a**-**h**: The wild type GFP and OsGCNT-GFP are separately transformed into rice protoplasts; **i**-**l**: Co-localization of OsGCNT-GFP with mcherry marker at GA; **m**-**p**: OsGCNT-GFP couldn’t co-localize with ER marker. Scale bars = 5 μm

Most GTs act along a secretory pathway requiring the endoplasmic reticulum and the Golgi body where GTs transfer monosaccharide units to various receptor molecules [34]. To determine the cellular distribution of OsGCNT, TargetP version 1.1 (http://www.cbs.dtu.dk/services/TargetP/) is first used to predict the subcellular localization, and we found that OsGCNT is predicted to be localized in the secretory pathway with the strongest confidence (reliability class (RC) 1). To determine the actual subcellular localization of OsGCNT, we then carried out a PEG-mediated transient expression of rice protoplasts transformed with OsGCNT-GFP. When pOsGCNT-GFP and pAN580 were introduced into the rice protoplasts, the distribution of OsGCNT-GFP was more pronounced in other locations compared to cytosol-localized GFP (Fig. 5). We confirmed the subcellular location of OsGCNT-GFP by expressing it with the Golgi marker, finding that the fusion protein was mainly localized to the Golgi apparatus (Fig. 5). Thus, we concluded that OsGCNT probably localized to the Golgi apparatus.

### *OsGCNT* indirectly regulates premature leaf senescence

**Fig. 6:**
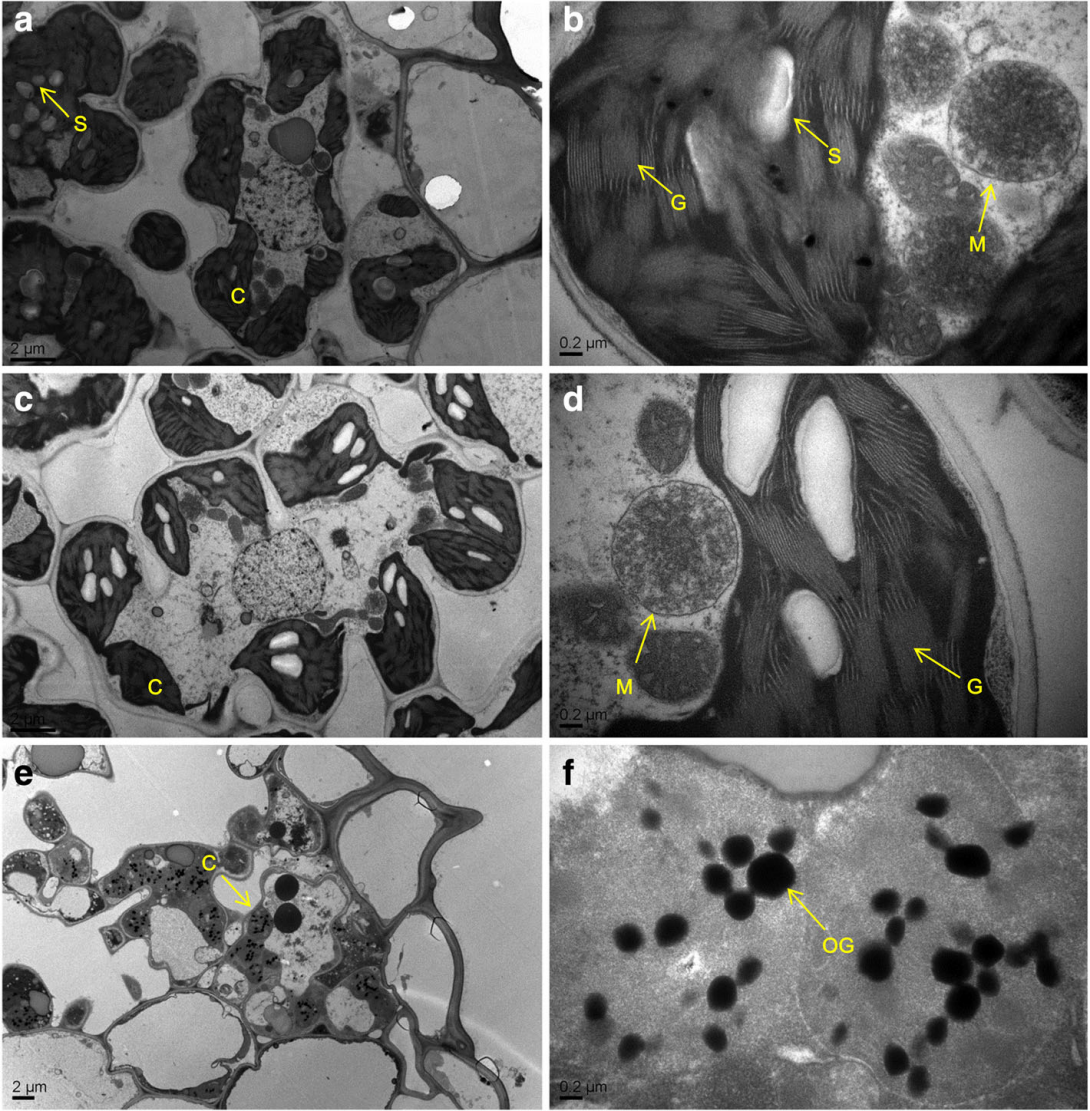
Ultrastructure of the chloroplast. **a** and **b** WT; **c** and **d** the green leaf of *spl21*; (**e**, **f**) the premature senescence leaf of *spl21*; C, chloroplast; M, mitochondrion; OG, osmiophilic granule; S, starch granule; G, grana thylakoid

It has been reported that some spl mutants develop spontaneous necrotic lesions accompanied by premature leaf senescence [23]. Decreased level chlorophyll content is used as a critical indicator for the presence of premature leaf senescence [15]. In our previous study, Chl a, Chl b and Car contents in *spl21* leaves were all significantly reduced compared to those of WT at different leaf developmental stages [33]. To further investigate the cause of the lower chlorophyll level in the *spl21* mutant during senescence, we compared the ultrastructure of chloroplasts in *spl21* and WT using transmission electron microscopy (TEM). The normal green leaves of *spl21* showed similar chloroplast morphology from mesophyll cells as the WT (Fig. 6a-d), that is, chloroplasts were well-developed with rich lamellae and a small number of osmiophilic bodies. However, disordered structure of thylakoid grana and stroma lamella and degenerated thylakoid membrane were observed in the premature senescent leaves of *spl21* (Fig. 6e, f). These results suggested that the mutation of *OsGCNT* significantly affected the breakdown of chloroplasts in *spl21*.

**Fig. 7:**
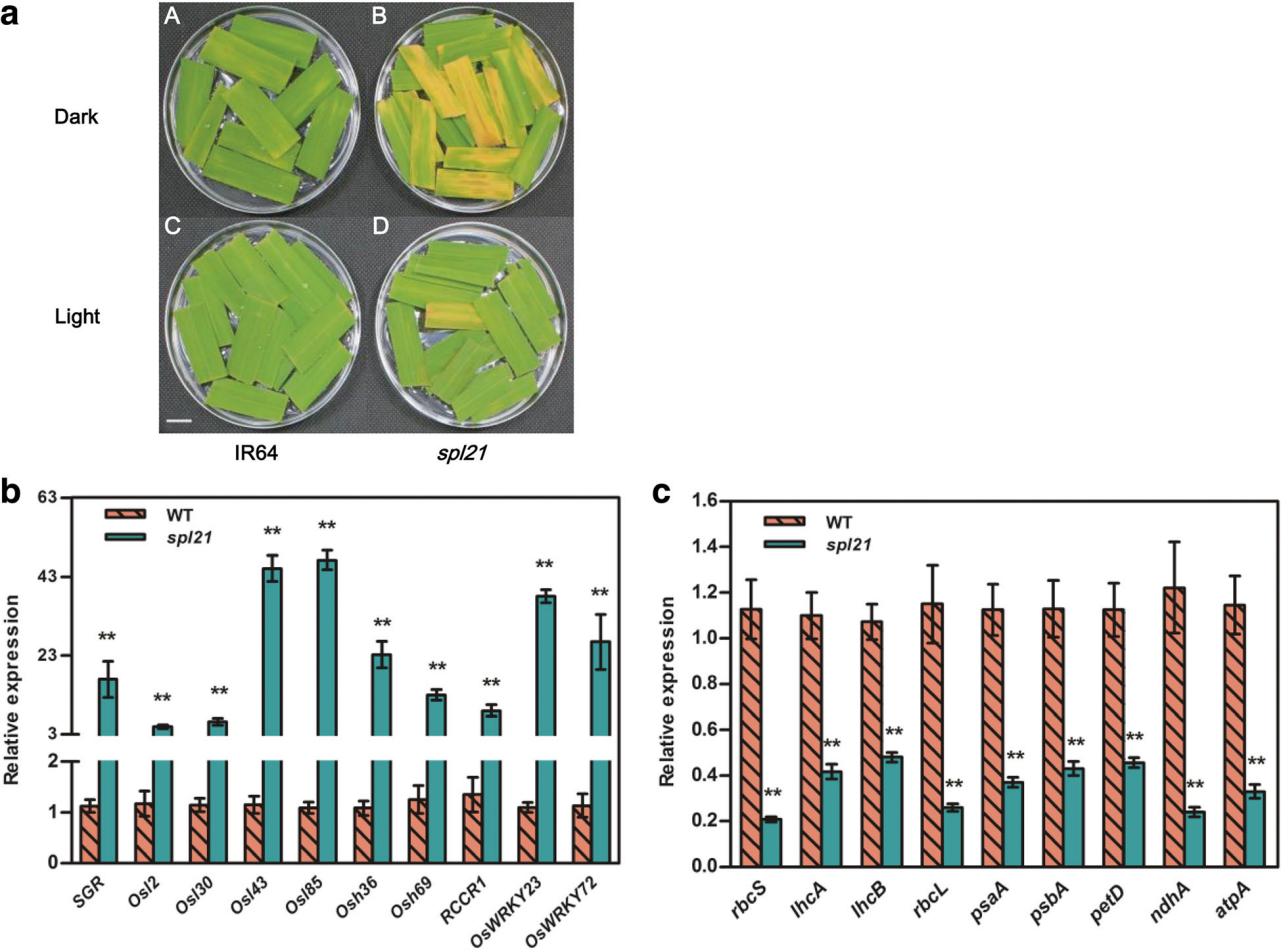
Darkness-induced leaf senescence and gene expression analysis. **a** Detached flag leaves from IR64 and *spl21* at the heading stage were incubated with normal light (H_2_O) and darkness (H_2_O) at 30 °C for 5 d. Scale bar = 1 cm. **b** Expression profile of senescence genes in IR64 and *spl21* at heading stage. **c** Expression profile of photosynthesis-related genes in IR64 and *spl21* at heading stage; the expression level of each gene in IR64 was normalized to 1. Error bars in (**b**, **c**) indicate standard deviations of three independent samples. Data are means ± SD of three biological replicates. Single asterisk denotes *P* ≤ 0.05, and double asterisks denote *P* ≤ 0.01 (Student’s *t*-test)

It is well known that leaf senescence is highly associated with various environmental stresses such as darkness [35]. Consequently, darkness treatment is performed frequently to induce synchronous senescence in plants [36]. To reveal whether leaf senescence progress of *spl21* could be accelerated in darkness, we performed darkness treatment on the detached flag leaves from *spl21* and WT. After incubation in darkness and light control conditions for five days, the *spl21* leaves exhibited yellowing phenotype while the wide type IR64 leaves remained greener under darkness conditions (Fig. 7a). Thus, we concluded that *spl21* leaf senescence progress could be accelerated in darkness.

Leaf senescence is a complex biological process controlled by induced expression of a large number of relevant genes [37]. Among them, a senescence-induced gene, *STAY GREEN* (*SGR*), has been reported to regulate chlorophyll degradation [38]. Expression levels of *SGR* revealed that *SGR* transcripts were dramatically upregulated in the *spl21* mutant at the heading stage (Fig. 7b). It has been shown that the expression levels of many senescence-associated genes (SAGs) and transcription factors (TFs) in spl mutants are upregulated during premature leaf senescence [3]. To further prove that leaf senescence occurred in *spl21*, we determined the expression of 6 SAGs (*Osl2*, *Osl30*, *Osl43*, *Osl85*, *Osh36* and *Osh69*), one chlorophyll degradation-related gene (RCCR1), and 2 senescence-associated TFs (*OsWRKY23*, *OsWRKY72*) in *spl21* and WT at the heading stage, respectively. The results demonstrated that the expression levels of all nine genes were notably increased in the mutant at heading stage in comparison with WT (Fig. 7b), in agreement with the symptom of leaf senescence, indicating that premature leaf senescence of *spl21* was associated with the up-regulation of SAGs.

Leaf senescence is also accompanied by the lower expression of genes related to photosynthesis [7]. We assayed the expression levels of photosynthesis-associated genes including *rbcS*, *lhcA*, *lhcB*, *rbcL*, *psaA*, *psbA*, *petD*, *ndhA* and *atpA* (Fig. 7c). qRT-PCR analysis showed that the mRNA levels of 3 nuclear-encoded genes (*rbcS*, *lhcA*, *lhcB*) and 6 chloroplast-encoded genes (*rbcL*, *psaA*, *psbA*, *petD*, *ndhA*, and *atpA*) were significantly down-regulated in *spl21* leaves. These molecular evidences indicated that the *OsGCNT* mutation was responsible for the premature leaf senescence of *spl21*.

### *OsGCNT* regulates defense response

**Fig. 8:**
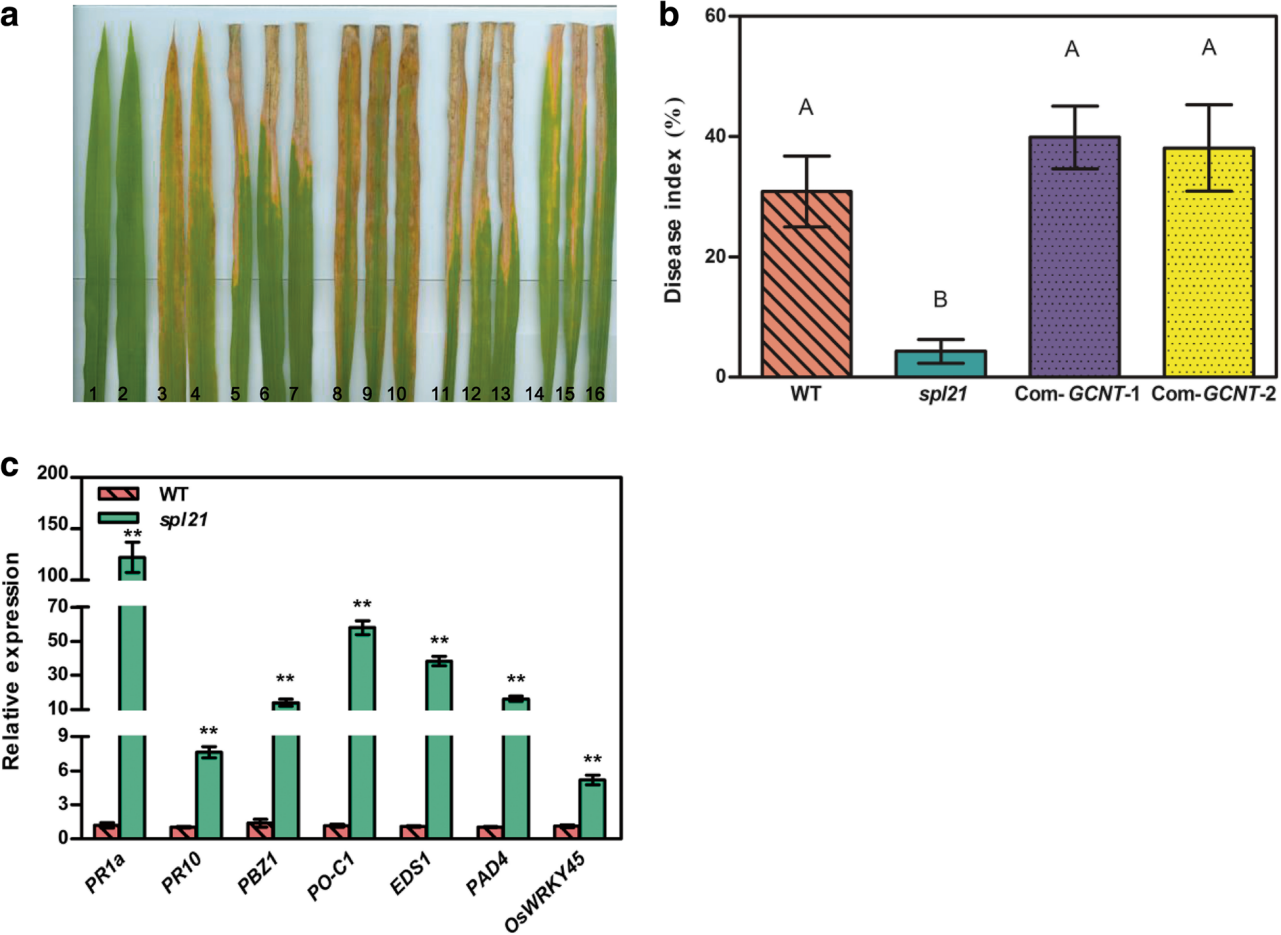
Evaluation of disease resistance and expression of defense signaling-related genes in *spl21* and WT. **a** Reactions to *Xoo* race PXO99. 1–2: WT before inoculation; 3–4: *spl21* before inoculation; 5–7: WT after inoculation; 8–10: *spl21* after inoculation; 11–16: complementation lines. **b** Mean lesion length/leaf length radio after inoculation of plant leaves with PXO99. Data represent the lesion length means ± SD from 5 independent plants at the tillering stage (Student’s *t*-test: **, *P* ≤ 0.01). **c** Relative expression of seven defense signaling-related genes in WT and *spl21* at the tillering stages analyzed by qRT-PCR. The expression level of each gene in WT was normalized to 1; data represent the mean ± SD of three biological replicates (Student’s *t*-test: **, *P* ≤ 0.01)

The typical phenotype of numerous spl mutants is similar to that of HR in plants following infection by invading pathogens [2]. To estimate disease resistance to *Xanthomonus oryzae* pv. *oryzae* (*Xoo*), we inoculated *spl21*, WT and complemented plants with the virulent *Xoo* strain PXO99 at the tillering stage. The WT plants and two complemented plants showed longer lesions to PXO99 while the *spl21* plants exhibited a significantly enhanced resistance with shorter lesions on the leaf blades (Fig. 8a, b).Since the differential response patterns of *spl21*, WT and complemented plants to bacterial blight infection might indicate that the plant systemic resistance to diseases was obviously changed in these lines, we measured the expression levels of 7 defense-response marker genes (*PR1a*, *PR10*, *PBZ1*, *PO-C1*, *EDS1*, *PAD4* and *OsWRKY45*) at the tillering stage using relative quantification by qRT-PCR. Our results showed that the mRNAs levels of *PR1a*, *PR10*, *PBZ1*, *PO-C1*, *EDS1*, *PAD4* and *OsWRKY45* in *spl21* were highly increased by 121.9-, 7.6-, 13.9-, 57.9-, 38.3-, 16.2-, 5.2-fold, respectively, compared to that of WT (Fig. 8c), indicating that the enhanced level of disease resistance was accompanied by the up-regulation of defense response genes.

### Transcriptome sequencing suggests that *OsGCNT* regulates defense response

To further explore the global effects of the *OsGCNT* mutation which could enhance disease resistance and immunity-associated premature leaf senescence of the *spl21* mutant, we analyzed gene expression profiles of *spl21* and WT by high-throughput RNA sequencing (RNA-seq). Based on the RNA-seq data, 4315 DEGs were revealed in the *spl21* mutant, including 2454 up-regulated and 1861 down-regulated ones.

GO analysis was conducted to classify the DEGs identified in *spl21* at the functional level. All the reliable DEGs were analyzed for GO functional enrichment. From the total GO functional enrichment results, functional categories including oxidation-reduction process (GO: 0055114), catalytic activity (GO: 0003824) and kinase activity (GO: 0016301) were significantly enriched in *spl21* (Additional file 1: Dataset 1, total group). Notably, enzymatic activity genes involved in response to oxidation-reduction process (GO: 0055114), oxidoreductase activity (GO: 0016491), monooxygenase activity (GO: 0004497), and tetrapyrrole binding (GO: 0046906) were significantly enriched among the 2454 up-regulated genes in *spl21* (Additional file 1: Dataset 1, up-regulated group). On the other hand, 1861 down-regulated genes were significantly enriched on photosynthesis (GO: 0015979) and single-organism biosynthetic process (GO: 0044710) (Additional file 1: Dataset 1, down-regulated group). These results demonstrated that *OsGCNT* might be also involved in photosynthesis and oxidation-reduction processes in rice.

For further investigations of the biological pathways associated with *OsGCNT*, we mapped the DEGs in the KEGG database and performed enriched pathways between *spl21* and WT. The results indicated that the ‘biosynthesis of secondary metabolites’ (ID: osa01100) was verified as one of the most significantly enriched pathways (Additional file 2: Dataset 2). There were seven pathways significantly enriched for up-regulated genes, and the highly enriched pathways were biosynthesis of secondary metabolites, metabolic pathways and phenylalanine metabolism (Additional file 2: Dataset 2, up-regulated group). The ‘metabolic pathways’ (ID: osa01100), ‘endocytosis’ (ID: osa04144) and ‘photosynthesis’ (ID: osa00061) were highly enriched pathways in down-regulated genes (Additional file 2: Dataset 2, down-regulated group). These results indicated that *OsGCNT* was likely involved in biosynthesis of secondary metabolites, and regulation of defense response.

Among the 4315 DEGs, 28 genes were involved in jasmonate signaling pathway including 18 jasmonate synthesis/degradation-associated genes, 5 WRKY genes, 3 phenylalanine ammonia-lyase (PAL) genes and 2 jasmonate induced-regulated genes (Additional file 3: Table S1). 20 out 28 genes including 10 cytochrome P450-econding genes, 2 WRKY, 3 PAL, *OsJAZ9*, *OsOPR1*, *OsSL* were upregulated by 2.4–50.1 folds while the other remaining 8 genes were down-regulated by 2–28.1 folds. To validate, we chose 10 JA signaling pathway-associated genes for qRT-PCR analysis. Our qRT-PCR results showed that the mRNAs levels of *OsLOX1*, *OsJAZ9*, *OsOPR1*, *OsSL OsWRKY42, OsWRKY48, OsPAL2*, *OsPAL5* and *OsPAL7* in *spl21* were highly increased by 5.7-, 9.0-, 38.1-, 30.3-, 22.5-, 7.5-, 5.0-, 5.8-fold, respectively, compared to those of WT (Additional file 4: Figure S1). In contrast, the expressions of *OsLOX7* and *OsWRKY82* were down-regulated by 2.4- and 2.3-fold compared with WT, respectively. These results were consistent with those of transcriptome sequencing results. Thus, we illustrated that the JA-mediated signaling pathway was probably constitutively activated in *spl21*.

**Fig. 9:**
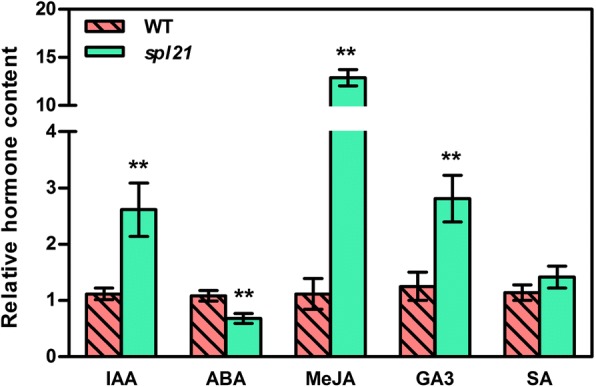
Hormone levels in leaves of *spl21* and WT. IAA, indole acetic acid; ABA, abscisic acid; MeJA, jasmonate; GA3, gibberellin A3; SA, salicylic acid. Data represent means ± SD of three biological replicates. Single asterisk denotes *P* ≤ 0.05, and double asterisks denote *P* ≤ 0.01 (Student’s *t*-test)

To investigate whether the gene expression alteration is associated with the content of endogenous plant hormones including MeJA, SA, IAA, GA3 and ABA, we measured the hormone levels of WT and *spl21* at the tillering stage. The results showed that the levels of MeJA, IAA, and GA3 in *spl21* were 11.5-, 2.34-, 2.25-fold, respectively, compared to the levels of WT, whereas the level of ABA decreased by 34.3% (Fig. 9). These results suggested that the enhanced disease resistance of *spl21* was probably resulted from the activation of the JA-mediated signaling pathway and inactivation of ABA-mediated signaling pathway.

## Discussion

### Functional inactivation of *OsGCNT* is responsible for the spotted-leaf phenotype

In the present study, we demonstrate that the mutant GCNT allele has a single nucleotide replacement (A836G) leading to the amino acid substitution (Y279C) in the translated protein (Fig. 1). Functional Complementation with the wild type allele could rescue the phenotype in *spl21* (Fig. 2). The amino acid sequence alignment indicates that GCNT proteins contain a Branch domain conserved in a wide range of organisms (Fig. 3). There are two types of *beta*-1,6-N-acetylglucosaminyl transferase, I-branching GCNT and core-2 branching GCNT. These GCNTs display different biological functions as the I-branching enzyme could produce the blood group I-antigen while core-2 branching enzyme constitutes the crucial side-chain branches in O-glycans [39, 40]. RNA-seq analysis of *spl21* and WT demonstrated that both ‘catalytic activity’ GO terms in molecular function and ‘biosynthesis of secondary metabolites’ in KEGG pathways were significantly higher enriched (Additional file 1: Dataset 1 and Additional file 2: Dataset 2). These results indicate that OsGCNT may be involved in catalyzing the modification of biological molecules, the typical role of glycosyltransferase in biosynthetic processes. Therefore, we assume that OsGCNT is likely to fulfill the functions of *beta*-1,6-N-acetylglucosaminyl transferase. However, further investigation is needed to distinguish which type of *beta*-1,6-N-acetylglucosaminyl transferase to which OsGCNT belongs.

*OsGCNT* is constitutively expressed in all tissues and expressed at all developmental stages in the WT plants (Fig. 4). The subcellular location of OsGCNT shows that OsGCNT-GFP mainly localizes to the Golgi apparatus in rice (Fig. 5). In addition, ‘vesicle’, ‘cytoplasmic membrane-bounded vesicle’ and ‘cytoplasmic vesicle’ GO terms in cellular component are highly enriched within the up-regulated genes (Additional file 1: Dataset 1, up-regulated group). It is known that the plant Golgi apparatus consists of individual stacks of membrane bound flattened cisternae surrounded by small vesicles that are dispersed throughout the cytoplasm [41].Thus, our results imply that OsGCNT may involve in transportation of cellular vesicles. It has been shown that N-acetylglucosaminyltransferase I (GCSI) from Arabidopsis localizes to the ER, while ß-1,2-xylosyltransferase is exclusively targeted to Golgi apparatus [42]. Furthermore, the human GCNT1 and GCNT2 both locate to the Golgi apparatus [43]. These relevant evidences and subcellular location of the fusion protein OsGCNT-GFP in the present study support that OsGCNT localizes to the Golgi apparatus. However, we have not yet tested whether OsGCNT-GFP is a functional version that can complement the *spl21* phenotype. Therefore, the actual intracellular location of OsGCNT till now is not certain and needs to be elucidated in the future.

The single nucleotide replacement (A836G) causes the amino acid substitution (Y279C) in the OsGCNT protein sequence and is responsible for the defective phenotype in *spl21*. To the best of our knowledge, this is the first report of a rice *beta*-1,6-N-acetylglucosaminyl transferase mutant which displays a typical spotted-leaf phenotype.

### Loss-of-function of *OsGCNT* induces innate immunity and immunity-associated premature leaf senescence

The *spl21* senescence symptoms appear at the four-leaf stage seedlings based on our previous study [33]. In recent years, several studies have shown that the premature leaf senescence is accompanied by the spotted-leaf phenotype although the mechanisms underlying premature leaf senescence has not been fully clarified in these mutants [3, 21, 24]. Consequently, it is important to confirm that the premature leaf senescence does really occur in *spl21* using senescence indicators including the decrease of chlorophyll content [15], chloroplast breakdown [24], dark-induced senescence [35], up-regulation of senescence-associated genes [3, 38, 44, 45], and down-regulation of photosynthesis-related genes [21]. We found that chloroplast degradation in *spl21* (Fig. 6) was coincident with the premature leaf senescence process similar to rice *spl128* [24]. Senescence-associated genes such as chlorophyll degradation regulated gene (*SGR*) [38], breakdown of chlorophyll metabolism-related gene (*RCCR1*) [44], senescence-associated transcription factors (*OsWRKY23* and *OsWRKY72*) [45], and senescence associated genes *Osl2*, *Osl30*, *Osl43*, *Osl85*, *Osh36*, *Osh69* [3], were significantly up-regulated in *spl21* (Fig. 7b). Furthermore, a set of photosynthesis-related genes were prominently down-regulated in *spl21* (Fig. 7c). All these evidences support that premature leaf senescence occurs in *spl21*. We therefore conclude that *OsGCNT* participates indirectly in the regulation of leaf senescence.

Many spl mutants exhibit the upregulated expression of defense response genes and spontaneous appearance of HR-like lesions, both of which may contribute to enhanced resistance to pathogens [5]. In our present study, we demonstrate that the *spl21* mutant acquired systemic resistance to compatible strains of rice bacterial leaf blight pathogen compared to both WT and complementary plants (Fig. 8a, b), suggesting that *OsGCNT* serves as a negative regulator of defense response in rice. Enhanced defense response can also be reflected by the up-regulation of defense response-associated genes such as *PR1a*, *PR10*, *PBZ1*, *PO-C1*, *EDS1*, *PAD4* and *OsWRKY45* which have commonly been used as molecular markers for rice defense response [14, 19]. As expected, the expression level of defense response-associated genes (*PR1a*, *PR10*, *PBZ1*, *PO-C1*, *EDS1*, *PAD4* and *OsWRKY45*) were all increased remarkably in *spl21* after the initiation of leaf lesions (Fig. 8c), suggesting that *OsGCNT* is involved in enhancing defense response. Whether defense response occurs before the initiation of lesions remains to be investigated.

### JA may play an important role in innate immunity and immunity-associated senescence in *spl21*

Plant small-molecule hormones such as SA, JA, and ABA play important regulatory roles in most biological processes of plants, including promote or delay of the leaf senescence and up-, down-regulate of defense response genes [7]. MeJA acts as dominating cellular regulator modulating leaf senescence in JA signaling pathways [46]. In the present study, endogenous MeJA levels were significantly elevated in *spl21* (Fig. 9). It has been shown that MeJA could cause rapid chlorophyll loss and induce the expression of several key enzymes involved in chlorophyll breakdown [47], and furthermore, exogenous application of MeJA induces leaf senescence and increases the accumulation of secondary metabolites in various plant species [48, 49]. Furthermore, MeJA-induced senescence is not associated with the level of endogenous ABA in detached rice leaves [50]. These results suggest that the premature leaf senescence in *spl21* is mainly modulated by the increased level of MeJA while it is still essential to further investigate the molecular mechanism underlying leaf senescence mediated by endogenous MeJA level.

Furthermore, consistent with the higher endogenous MeJA content (Fig. 9), multiple genes involved in response to JA were activated in *spl21* (Additional file 3: Table S1, Additional file 4: Figure S1), supporting the notion that resistance to *Xoo* was enhanced via the JA-mediated pathway. It has been identified that the 12-oxo-phytodienoic acid reductase gene *OsOPR1* encoded the last committed enzymatic step on the octadecanoid pathway leading to jasmonic acid (JA) biosynthesis [51]. Transcription of the *OsOPR1* is shown to be JA-inducible and the increase of *OsOPR1* mRNA levels by JA does not appear to require de novo synthesis of proteins [51]. Increased expressions of pea *LOXs* have been identified along with the release of signal molecules like MeJA [52]. In rice, *CYP450* enzyme is proved to be involved in JA signaling pathway [53]. *OsJAZ9* has been proved to be involved in JA signaling pathway acting as a transcriptional regulator modulating salt stress tolerance in rice [54]. Considering the key role of JA in plant disease resistance, we therefore reason that the highly accumulated MeJA in *spl21* is probably involved in OsGCNT-mediated defense response although the molecular mechanism underlying the resistance to *Xoo* remains elusive.

## Conclusions

In the present study, we reported the map-based cloning of a novel gene *Oryza sativa beta-1,6-N-acetylglucosaminyl transferase* (*OsGCNT*) responsible for the spotted-leaf phenotype of *spl21*. The loss of function of OsGCNT induced enhanced disease resistance to *Xoo* in *spl21*. The novel OsGCNT, acting as a negative regulator, modulated rice innate immunity and immunity-associated leaf senescence probably by activating the jasmonate signaling pathway.

## Methods

### Plant materials

The spotted-leaf mutant *spl21* was isolated from an ethane methyl sulfonate (EMS) mutagenesis of *indica* rice cultivar IR64 [5]. The mutant phenotype, spotted leaf and leaf senescence, has been stably inherited over multiple generations. The rice *spl21* shows an impaired growth and spotted-leaf lesions from the four-leaf stage to the ripening stage. Furthermore, agronomic traits, such as plant height, number of tillers/plant, seed-setting rate, and 1000-grain weight, were significantly decreased in the *spl21* mutant in contrast to the wild-type (WT) IR64 [33]. *spl21* and WT were planted both in the greenhouse and the paddy field under normal water and fertilizer management at the China National Rice Research Institute (CNRRI) in 2017.

### Complementation test

To construct a vector for functional complementation, an 5557 bp DNA genomic fragment from WT containing 1619 bp upstream of the *OsGCNT* transcription start site, the full-length *OsGCNT* genomic DNA, and 1473 bp downstream of the *OsGCNT* termination site was amplified by LA taq polymerase (Takara *Inc.*) using the primer set 28com (Additional file 5: Table S2). The 5557 bp fragment was then cloned into the binary vector pCAMBIA1300 to generate a new construct pC1300-C. The construct was used to transform embryogenic calli induced from *spl21* seeds using the *Agrobacterium tumefaciens-*mediated method [55]. Positive transgenic plants were confirmed using Bar178 primers (Additional file 5: Table S2) specific for the amplification of the phosphinothricin screening gene.

### Multiple sequence alignment

The Rice Genome Annotation Project (http://rice.plantbiology.msu.edu/) and the Simple Modular Architecture Research Tools program (http://smart.embl-heidelberg.de/) were respectively used to predicted gene and the major functional domains of OsGCNT. The NCBI Blastp search program (http://www.ncbi.nlm.nih.gov/) was used to search protein homologous sequences to OsGCNT. The sequences of GCNT were aligned using DNAMAN v6.0 (http://www.lynnon.com/) and the neighbor-joining tree was conducted using the software MEGA v7.1 (http://www.megasoftware.net/).

### RNA extraction and quantitative real-time PCR analysis

Total RNA was extracted from various plant tissues using NucleoZOL Reagent Kit according to the manufacturer’s protocol (MACHEREY-NAGEL GmbH & Co. KG). The genomic DNA removal and the first stand cDNA synthesis were carried out using the ReverTra Ace qPCR RT Master Mix Kit (Toyobo, Japan). Fast Star Essential DNA Green Master Kit (Roche, Switzerland) was chosen for qRT-PCR analysis and performed on a Thermal Cycle Dice® Real Time System (TaKaRa, Japan). An *ubiquitin* gene (*LOC_Os03g13170*) was used as an internal control. Primers used for qRT-PCR are listed in Additional file 5: Table S2. Three replicates were used for each biological sample and the means were used for analysis.

### Subcellular localization of OsGCNT protein

The full-length coding sequence (no stop codon) of *OsGCNT* was amplified using the primers SPL21-GFP F/R (Additional file 5: Table S2). The PCR product of *OsGCNT* was fused to the GFP N-terminus and driven by the CaMV 35S promoter in the transient expression vector PAN580 to generate a new construct designated as pOsGCNT-GFP. The construct was co-transformed in rice protoplasts with the marker plasmid ST-RFP [56], and transfected protoplasts were incubated as described previously [57]. The GFP fluorescence was observed 48 h after transformation by a Zeiss lsm710 confocal laser scanning microscope (Carl Zeiss, Inc., Jena, Germany).

### Analysis of pigment and transmission electron microscopy

Leaf samples from *spl21*, WT and complemented plants were used to determine contents of Chl a, Chl b, Chl T and Car at the tillering stage following the method of Wellburn [58]. Leaves of the control plants at the same stage were used for comparison analysis. Transverse sections of leaves from WT and *spl21* leaves with lesions were used for transmission electron microscopy observation according to the method described previously [59].

### Dark-induced senescence

At the heading stage, the flag leaves were cut into ~ 3 cm pieces and floated on 20 mL of distilled water in Petri dishes. The leaf samples were incubated at 30 °C in darkness and normal light for 5 days in a growth chamber with daily cycles of 10 h of light and 14 h of darkness.

### Disease resistance evaluation

The virulent *Xanthomonas oryzae* pv. *oryzae* strain PXO99 (Philippine race 6) was routinely cultured in a peptone sucrose agar medium. A total of 6–8 leaf blades including the upper-most fully expanded leaves from both the main and lateral tillers of WT, *spl21* and 2 complemented plants were inoculated with a bacterial suspension (OD_600_ = 1.0) using the leaf clipping method [60]. Lesion length was measured 12 days after inoculation. The lesion length means of 5 leaf blades were used for analysis.

### Hormone level determination

The levels of indole-3-acetic acid (IAA), methyl jasmonate (MeJA), gibberellin 3 (GA3), salicylic acid (SA) and abscisic acid (ABA) at the tillering stage were determined by Zoonbio Biotechnology Co., Ltd., following the methods described previously by Zhang et al. [27]. The means of three replicates were used for analysis.

### Transcriptome sequencing and data analysis

To eliminate other possible mutations, the BC_2_F_3_ population was derived from the cross between *spl21* mutant and WT IR64. Top-second leaves from 3 individual BC_2_F_3_ mutant plants and 3 individual WT plants at heading stage were used for RNA isolation after grinding in liquid nitrogen. A total of 6 RNA samples were used for RNA-seq analysis and Q-RT-PCR conformation. The RNA samples were quantified using an Agilent 2100 Bioanalyzer system (Agilent Technologies, Waldbronn, Germany) before sequencing analysis. High-throughput sequencing was performed on an Illumina HiSeq 3000 platform (Illumina, San Diego, CA, USA) following the manufacturer’s recommendations. The aligned read files were processed by Cufflinks [61]. The unit of measurement is Fragment Per Kilobase of exon per Million fragments mapped (FPKM). Differentially expressed genes (DEGs) and transcript expression analysis between three biological replicates of either *spl21* and IR64 were performed using the Empirical Analysis of Digital Gene Expression data package in Cuffdiff (ver.2.1.1). An absolute fold change of > 2 and a False Discovery Rate (FDR) significance score of ≤0.05 were used as thresholds to identify significant differences in gene expression. Gene Ontology (GO) annotations of DEGs were obtained from the Rice Genome Annotation Project. GO functional enrichment and Kyoto Encyclopedia of Genes and Genomes (KEGG) analysis were performed as described previously [62].

## Additional files

Additional file 1: Dataset 1. GO enrichment analysis of DEGs. (XLS 69 kb)

Additional file 2: Dataset 2. Functional enrichment analysis of DEGs based on KEGG metabolic pathways. (XLS 52 kb)

Additional file 3: **Table S1.** JA biosynthesis-associated genes between the IR64 and *spl21* plants in DEGs. (DOCX 23 kb)

Additional file 4: **Figure S1.** qRT-PCR validation of 10 genes expressed in IR64 and *spl21. (DOCX 340 kb)*

Additional file 5: **Table S2.** Primers used in this study. (DOCX 24 kb)

## Abbreviations

ABA: Abscisic acid
Car: Carotenoid
CAZy: Carbohydrate Active Enzyme
CDS: Coding sequence
Chl a: Chlorophyll a
Chl b: Chlorophyll b
DEG: Differentially expressed gene
EMS: Ethane methyl sulfonate
FDR: False discovery rate
GA3: Gibberellin 3
GCNT: Beta-1,6-N-acetylglucosaminyl transferase
GO: Gene Ontology
GT: Glycosyltransferase
HR: Hypersensitive response
IAA: Indole-3-acetic acid
JA: Jasmonic acid
KEGG: Kyoto Encyclopedia of Genes and Genomes
LMM: Lesion mimic mutant
MDA: Malonaldehyde
MeJA: Methyl jasmonate
PCD: Programmed cell death
qRT-PCR: Quantitative real-time reverse transcription-PCR
RNA-seq: High-throughput mRNA sequencing
SA: Salicylic acid
SAG: Senescence-associated gene
spl: spotted-leaf
TEM: Transmission electron microscopy
TF: Transcription factor
WT: Wild type
*Xoo*: *Xanthomonas oryzae* pv. *oryzae*
