# Supplementary material for: Functional inactivation of OsGCNT induces enhanced disease resistance to Xanthomonas oryzae pv. oryzae in rice
Source: BMC Plant Biol. 2018 Nov 1;18:264. doi: 10.1186/s12870-018-1489-9 (PMC6211509; doi:10.1186/s12870-018-1489-9)
Supplement: Supplementary file 3 — Table S1. JA biosynthesis-associated genes between the IR64 and spl21 plants in DEGs. (DOCX 23 kb) [file 12870_2018_1489_MOESM3_ESM.docx]

Supplemental Table 1**.** JA biosynthesis-associated genes between the IR64 and *spl21* plants in DEGs

| Gene locus | description | Log_2_Ratio  (*spl21*/IR64) | *P-*value | FDR |
| --- | --- | --- | --- | --- |
| *Os03g0700700* | OsLOX1; lipoxygenase, expressed | 1.94 | 5.00E-05 | 1.75E-04 |
| *Os03g0179900* | OsLOX2, lipoxygenase, putative, expressed | 1.31 | 5.00E-05 | 1.75E-04 |
| *Os05g0304600* | OsLOX7, putative, expressed | -1.30 | 5.00E-05 | 1.75E-04 |
| *Os02g0194700* | lipoxygenase, putative, expressed | -4.81 | 5.00E-05 | 1.75E-04 |
| *Os03g0180800* | OsJAZ9, Jasmonate ZIM-domain protein, TIFY domain-containing transcriptional regulator, expressed | 1.84 | 2.14E-02 | 4.12E-02 |
| *Os06g0216300* | OsOPR1; 12-oxophytodienoate reductase, expressed | 4.51 | 5.00E-05 | 1.75E-04 |
| *Os12g0268000* | OsSL; cytochrome P450 71A1, expressed | 3.05 | 5.00E-05 | 1.83E-02 |
| *Os01g0227400* | cytochrome P450, putative, expressed | 2.10 | 5.00E-05 | 1.75E-04 |
| *Os01g0543600* | cytochrome P450, putative, expressed | 5.66 | 1.15E-03 | 3.09E-03 |
| *Os01g0804400* | cytochrome P450, putative, expressed | 2.78 | 5.00E-05 | 1.75E-04 |
| *Os01g0804900* | cytochrome P450, putative, expressed | 2.57 | 4.45E-03 | 1.03E-02 |
| *Os03g0594100* | cytochrome P450, putative, expressed | 3.86 | 5.00E-05 | 1.75E-04 |
| *Os06g0641200* | cytochrome P450, putative, expressed | 2.42 | 5.00E-05 | 1.75E-04 |
| *Os07g0217600* | cytochrome P450, putative, expressed | 2.12 | 5.00E-05 | 1.75E-04 |
| *Os07g0518100* | cytochrome P450, putative, expressed | 2.16 | 5.00E-05 | 1.75E-04 |
| *Os07g0635200* | cytochrome P450, putative, expressed | 3.21 | 5.00E-05 | 1.75E-04 |
| *Os08g0547300* | cytochrome P450, putative, expressed | -3.98 | 5.00E-05 | 1.75E-04 |
| *Os02g0110200* | cytochrome P450, putative, expressed | -1.01 | 5.00E-05 | 1.75E-04 |
| *Os01g0750100* | OsWRKY13, expressed | 1.47 | 5.00E-05 | 1.75E-04 |
| *Os02g0462800* | OsWRKY42, expressed | 2.49 | 5.00E-05 | 1.75E-04 |
| *Os08g0276200* | OsWRKY82, expressed | -1.99 | 5.00E-05 | 1.75E-04 |
| *Os06g0649000* | OsWRKY28, expressed | -1.36 | 5.00E-05 | 1.75E-04 |
| *Os05g0322900* | OsWRKY45, expressed | -1.81 | 5.00E-05 | 1.75E-04 |
| *Os09g0249000* | kelch repeat-containing protein, putative, expressed | -1.32 | 4.50E-04 | 1.33E-03 |
| *Os07g0515000* | kelch repeat-containing protein, putative, expressed | 1.27 | 6.30E-03 | 1.41E-02 |
| *Os02g0626400* | OsPAL2, phenylalanine ammonia-lyase, expressed | 1.41 | 5.00E-05 | 1.75E-04 |
| *Os04g0518100* | OsPAL5, phenylalanine ammonia-lyase, expressed | 1.94 | 5.00E-05 | 1.75E-04 |
| *Os05g0427400* | OsPAL7, phenylalanine ammonia-lyase, expressed | 1.79 | 5.00E-05 | 1.75E-04 |

*P-*value, *P*-value from the differential gene expression test; FDR, false discovery rate, a method to determine the threshold of the *P-*value in multiple tests.
