# Supplementary material for: Functional inactivation of OsGCNT induces enhanced disease resistance to Xanthomonas oryzae pv. oryzae in rice
Source: BMC Plant Biol. 2018 Nov 1;18:264. doi: 10.1186/s12870-018-1489-9 (PMC6211509; doi:10.1186/s12870-018-1489-9)

Supplemental Figure 1. qRT-PCR validation of 10 genes expressed in IR64 and *spl21*

*Actin1* was used as an internal control. Double asterisks denote *P*-value < 0.01 (Student's *t*-test).


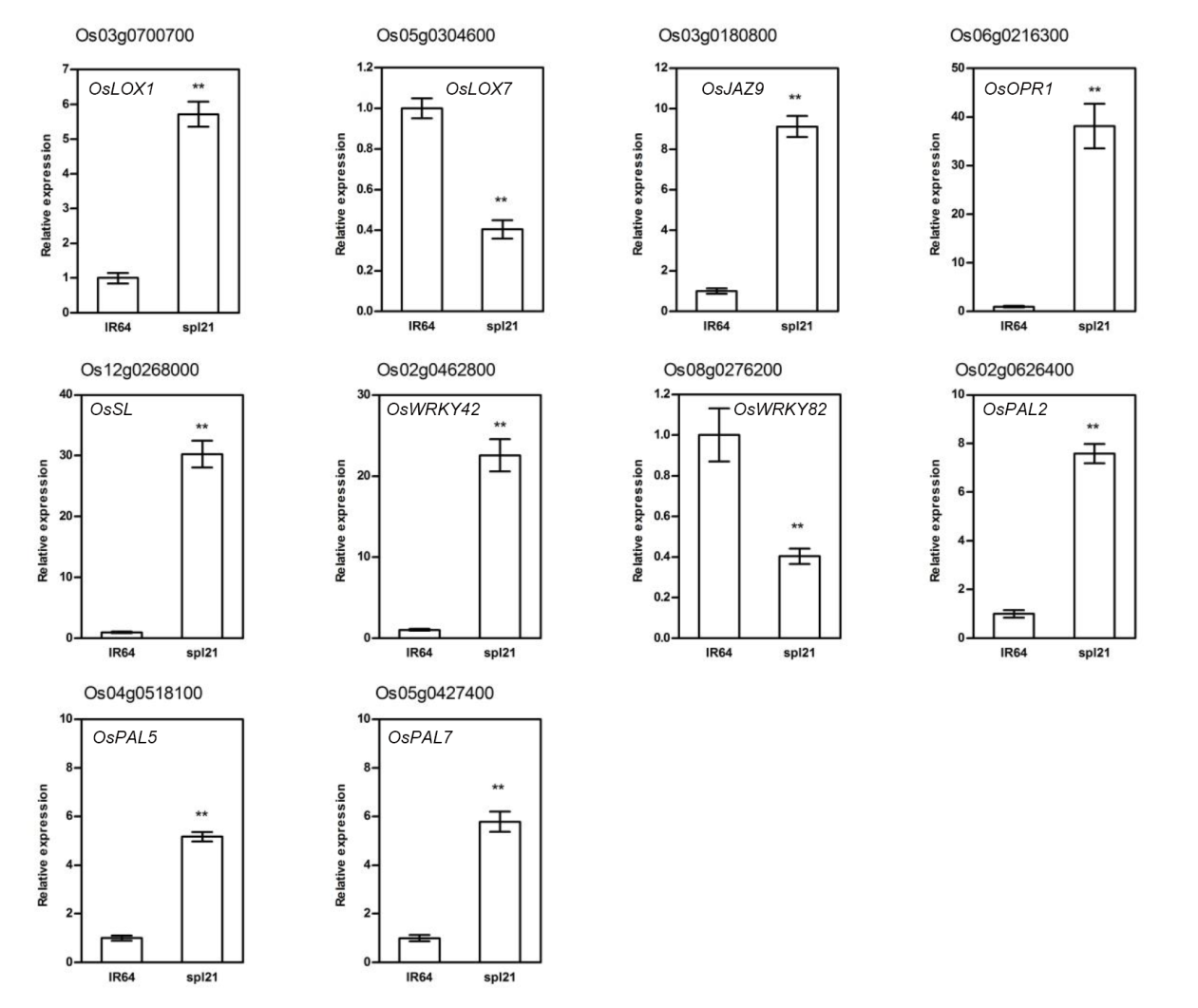

Supplement: Supplementary file 4 — Figure S1. qRT-PCR validation of 10 genes expressed in IR64 and spl21. (DOCX 340 kb) [file 12870_2018_1489_MOESM4_ESM.docx]
