# Supplementary material for: Functional inactivation of OsGCNT induces enhanced disease resistance to Xanthomonas oryzae pv. oryzae in rice
Source: BMC Plant Biol. 2018 Nov 1;18:264. doi: 10.1186/s12870-018-1489-9 (PMC6211509; doi:10.1186/s12870-018-1489-9)
Supplement: Supplementary file 5 — Table S2. Primers used in this study. (DOCX 24 kb) [file 12870_2018_1489_MOESM5_ESM.docx]

Supplemental Table 2. Primers used in this study

| Primers | Sequences (5′→3′) | Used for |
| --- | --- | --- |
| GCNT-COM | TCACAAACTGATGCTTCTGGAC  TCTCAACTGACCCAACCGT | Construction |
| GCNT-GFP | ACTAGTCGATTGCGTTGTTGTTCTTG  CCCGGGGTCTCGCAGGATGACGGAGT | Construction |
| InDel-8 | AAGCCGAAGGTTGGTTACTG  ATAGGGTTCTTATTGTTCAGCTCA | Mapping |
| *SGR* | GGCCTCCGCTACTACATCTT  TTGGAGTGGAAGTAGACCCA | q-PCR |
| *Osl2* | GCAGACAACAAATCGCCAAAT  TCTCCAGCAACTCTAACCAGCAT | q-PCR |
| *Osl30* | AACCTTTTTCTTGGAGATGATACAA  CTTGAACTGTAGGGGCTTGCTT | q-PCR |
| *Osl43* | TGTGACAAGTGCTAATAATACATACGA  CCAGACCTTCCAAAGAATCCAAC | q-PCR |
| *Osl85* | TCCAGGATGTGATGAGGATTATTC  GCGTGCTGTAGTTCAGTCTGTAAAG | q-PCR |
| *Osh36* | GTGCACCATGCACTTAATCC  CACCGACCCTTCCTGTAGTT | q-PCR |
| *Osh69* | TGCTGGATGGTGCAGAGTAGG  TCCGATGTGCGAGTAGACGAT | q-PCR |
| *RCCR1* | CGCATTTCCTCATGGAATTT  CTTCTCACGCTGTTTGTCCA | q-PCR |
| *OsWRKY23* | TCCAGTTCCTCTCCCAGTTCTAA  CACATTGTTCTCCTTTTCTTCCC | q-PCR |
| *OsWRKY72* | CACCACAAATCACATCTACTCCG  GCTGAAGGGAAGAGAGGTGAG | q-PCR |
| *OsACTIN1* | GCGTGGACAAAGTTTTCAACCG  TCTGGTACCCTCATCAGGCATC | q-PCR |
| *OsGCNT* | CCAATGGGACTCACTCCTAC  GCTTGTTCATCACATCTCAGC | q-PCR |
| *OsUbiquitin1* | CCCTCCACCTCGTCCTCAG  AGATAACAACGGAAGCATAAAAGTC | q-PCR |
| *rbcS* | TCATCAGCTTCATCGCCTAC  ACTGGGAACACACGAAACAA | q-PCR |
| *lhcA* | GTCTGTGGTTTGACCCGCT  GAGCCGCCCGTTCTTGA | q-PCR |

| *lhcB* | GCTCAAGGTGAAGGAGATCAAGA  ATGCGTTGTTGTTGACGGG | q-PCR |
| --- | --- | --- |
| *rbcL* | GTTGAAAGGGATAAGTTGA  AATGGTTGTGAGTTTACG | q-PCR |
| *psaA* | GAGATACCACTTCCTCAT  ACTAAGAAATTCTGCGTATT | q-PCR |
| *psbA* | AAGTTTCTCTGATGGTATG  ATAGCACTGAATAGGGAA | q-PCR |
| *petD* | TAATGGTTTCTGTGCCCACG  CCTAAAGTTAAGGATTTTTCAATGGG | q-PCR |
| *ndhA* | CAAAGCGATATCCCAAAGGAAT  CCGCATCGATACAACAACGTAT | q-PCR |
| *atpA* | GATCTCTCCAAACAGGCACAAG  CGGCTCTTTCTAAAAGGCGTG | q-PCR |
| *PR1a* | TGCATGTATGGACATGTAGTGTCATA  TACACTAAGCAAATACGGCTGACA | q-PCR |
| *PR10* | CACCATCTACACCATGAAGC  AGCACATCCGACTTTAGGAC | q-PCR |
| *PBZI* | CGCAAGTCATGTCCTAAAGTCG  ATGCCATAGTAGCCATCCACG | q-PCR |
| *PO-C1* | TCGGACCAGGTGCTCTTCAA  CTGATCTGGCCGTTTGTTCC | q-PCR |
| *EDS1* | CATTCCAAGAACGAGGACACTG  CAAGACTCAAGGCTAGAACCGA | q-PCR |
| *PAD4* | CCAACATGTACCGCATCAAG  GGTTGTTTCGGTGGTAGTGG | q-PCR |
| *OsWRKY45* | GAAGAATCATGGATGGACACGG  GACACATCAACAAGGAATTTACAAAC | q-PCR |
| *Os02g0626400* | GCATCAGCTTCCAACTCG  GGTTTCGCACTCCATTACAGA | q-PCR |
| *Os04g0518100* | TGCTGTCCGCCGTGTTCT  CCACCTCGTTCACCTTCTGC | q-PCR |
| *Os05g0427400* | CCAACCCTGTGACCAACCAT  GATCAAGAACGTCGAGGACATG | q-PCR |
| *Os03g0700700* | GATGGCGGTGCTCGACGTGCT  GCACCTGTTCTTGAGCTTTCTAT | q-PCR |

| *Os05g0304600* | CCGTCAACGATTCTGGG  GGTGAACCTCCAATGGG | q-PCR |
| --- | --- | --- |
| *Os03g0180800* | CGTCTCTCTTTGTGCTTGTG  ATCGTGAACTGCTGCCC | q-PCR |
| *Os06g0216300* | TCTCCACCACAGGTTCG  CAATCAAAGAGCACAATGACC | q-PCR |
| *Os12g0268000* | ATCGGCGACTTCTTCCC  CGTCTCCTTGATGATGGC | q-PCR |
| *Os02g0462800* | CAATTGACCGTTTGAATATTGG  TGAGGGAGGTAGCAGATCAA | q-PCR |
| *Os05g0583000* | AGTGAAAAGTAGTGAAAATTCCAG  GTGCTAGTTTCAATTATTCTGCTTCGT | q-PCR |
